# Complications in patients who have undergone laser refractive surgery

**Published:** 2024-10-02

**Authors:** Jie Ying, Aravind Roy, Prashant Garg, Wenxuan Chen

**Affiliations:** 1 Chair: Department of Ophthalmology, Tongren Hospital, Capital Medical University, Beijing, China.; 2 Consultant, Cornea and Anterior Segment Services, KVC Campus, L V Prasad Eye Institute, Vijayawada, India.; 3 Paul Dubord Chair of Cornea:, KAR Campus, L V Prasad Eye Institute, Hyderabad, India.; 4 MD: Department of Ophthalmology, Tongren Hospital, Capital Medical University, Beijing, China.

**Refractive surgery is a relatively safe procedure. Although complications are rare, being familiar with the signs and symptoms can support early detection and appropriate, timely referral and treatment.**

**Figure:**
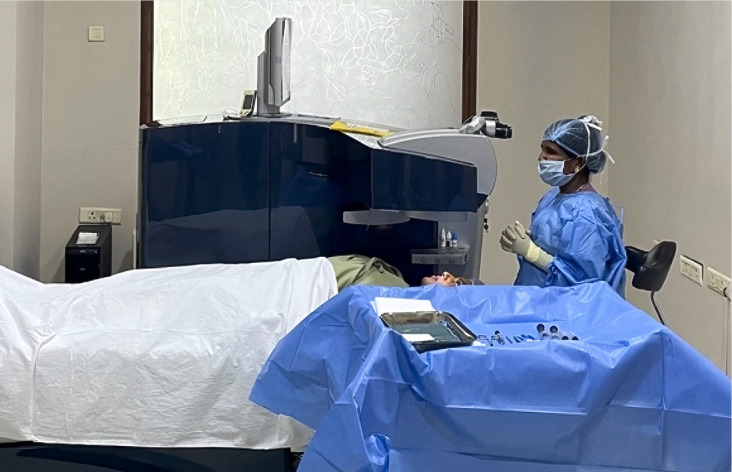
A patient is ready to undergo laser refractive surgery. INDIA

© ARAVIND ROY CC BY-NC-SA 4.0

Although refractive surgery is generally safe and effective, it is not free of complications. Due to its rising popularity, an increasing number of people are travelling long distances for refractive surgery and returning home for postoperative management; local eye care professionals may therefore be faced with any complications that occur. The aim of this article is to help you to recognise common complications after refractive surgery and guide management or referral, as appropriate.

Refractive surgery involves reshaping the cornea by cutting a hinged flap in the central part of the cornea, removing some corneal tissue, and then replacing the flap. There are different types of refractive surgery, such as laser-assisted in situ keratomileusis (LASIK), laser epithelial keratomileusis (LASEK), small incision lenticule extraction (SMILE), and photorefractive keratectomy (PRK). Complications can vary depending on the type, so it would be helpful to ask your patient if they know which they received.

## Dry eye disease

Dry eye disease manifests in the immediate postoperative period and is observed in over 90% of patients who have undergone corneal laser refractive surgery; it is particularly common in patients who have undergone laser-assisted in situ keratomileusis (LASIK) as the corneal nerves are cut. Dry eye symptoms are most bothersome in the first month after surgery and gradually improve over 3–12 months. Improvement in corneal sensation and dry eye disease occur by 3–6 months, but corneal re-innervation can be delayed by 2–5 years. Postoperative dry eye also predisposes patients to potentially sight-threatening complications such as refractive regression and microbial keratitis (see below).^1^

**Table 1:** Typical features of inflammatory and infective keratitis.

Clinical features	Inflammatory keratitis	Infection: microbial keratitis
Symptoms	No pain, redness, or watering Decreased vision when infiltrates involve the visual axis	Associated with pain, watering, redness, photophobia, and decreased vision if there is visual axis involvement
Ciliary congestion	Absent	Present
Corneal infiltrates	Whitish, may be diffuse (corneal stromal haze) There may be generalised corneal oedema	Yellowish, possibly dense, clustered area of infiltrates There may be dense corneal oedema, localised to a smaller area of the cornea
Anterior chamber inflammation	No cells or flare	Cells with or without flare in the anterior chamber; possible hypopyon
Microbiology (if available)	Negative	Positive for causative organisms, such as bacteria, fungi, Acanthamoeba, or Herpes simplex virus

### Symptoms

Ocular dryness, pain, stinging, photophobia, redness, visual fatigue, and fluctuating vision.

### Clinical signs

Decreased tear film stability, reduced tear secretion, fluorescein staining of the ocular surface, and laser-induced neurotrophic epitheliopathy.

### Management

At community or primary level, initial management consists of lubricating eye drops, warm compresses, and lid hygiene. If this does not address symptoms, patients need to be managed by an ophthalmologist who can prescribe different lubricant drops, topical cyclosporine, and slow tapering of topical steroids. Temporary punctal plugs can also be considered in patients with severe dry eye disease. See the article on dry eye disease in this issue for more information and guidance.

## Keratitis

Keratitis is relatively uncommon, but it is a potentially sight-threatening complication of refractive surgery procedures.^2^

**Figure 1:**
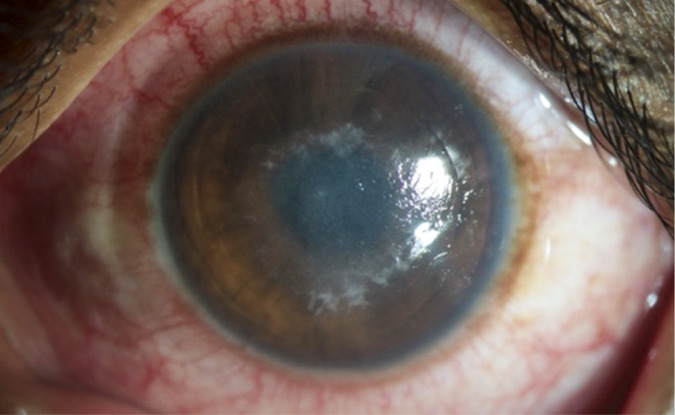
Microbial (infectious) keratitis with ciliary congestion, central corneal infiltrates, and irregular margins.

**Figure 2:**
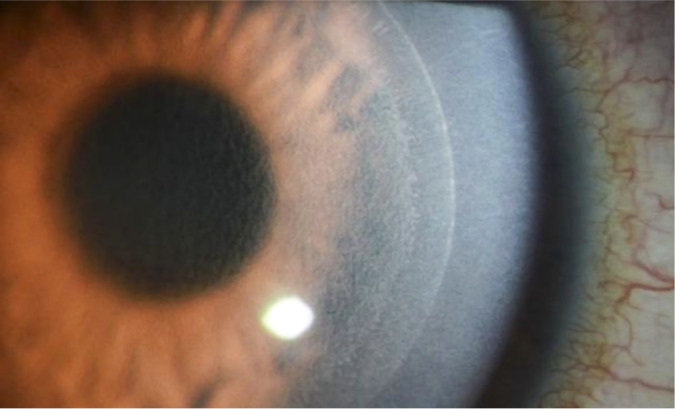
Diffuse lamellar keratitis starting at the periphery of the LASIK flap and progressing to the centre of the visual axis.

It is important to distinguish **microbial (infective) keratitis** (Figure 1, overleaf) from **inflammatory (sterile)** **keratitis**. The most common type of inflammatory keratitis after refractive surgery is **diffuse lamellar keratitis** (Figure 2), which normally manifests as infiltrates in the LASIK interface. This typically starts at the periphery of the flap and progresses along the interface, towards the visual axis. Table 1 compares the typical clinical features of these two types.

© VINEET JOSHI CC BY-NC-SA 4.0

© VINEET JOSHI CC BY-NC-SA 4.0

### What to ask the patient

- Ask about pain and photophobia – these are typical of microbial keratitis
- Ask about adherence with postoperative steroid medications. Non-adherence can increase the risk of diffuse lamellar keratitis
- Ask about the type of surgery, if they know – diffuse lamellar keratitis is associated with LASIK and SMILE.

### Management

If **infection** is suspected, start broad-spectrum antibiotics immediately. Refer the patient to an eye clinic if symptoms worsen or do not improve in 48 hours, ideally somewhere that can carry out a full microbiology workup.

If **inflammation** is suspected, continue topical corticosteroids. If infiltrates are worsening and/or involve the visual axis (leading to decreased vision), refer the patient to a corneal specialist for flap lift and interface wash.

**NOTE:** Corneal stromal haze that does not clear with increased doses of topical corticosteroids should alert you to the possibility of pressure-induced stromal keratitis (see the section on **increased intraocular pressure**). The treatment of pressure-induced stromal keratitis is to reduce the steroid drops, rather than maintaining or increasing the steroid drops, as you would with diffuse lamellar keratitis.

## Post refractive surgery ectasia

Post refractive surgery ectasia involves changes to the shape of the cornea, similar to what is seen in keratoconus, as a result of refractive surgery. It may manifest months to years after laser vision correction and is relatively rare, occurring in between 0.04 and 0.5% of patients, most commonly those who are young and have high myopia.

### Symptoms

Typically presents as blurred vision and a progressive increase of the refractive error.

### Clinical signs

See the article on keratoconus in this issue.

### Management

- Counsel the patient and advise them to avoid eye rubbing
- Consider using rigid gas permeable contact lenses to improve vision
- Consider collagen cross linking (see the article in this issue) and/or (less commonly) inserting intracorneal stromal ring segments.

Avoid further refractive surgical procedures in patients who have abnormal corneal tomography suggestive of ectasia.

## Flap striae

Flap striae are commonly seen after LASIK. If seen within the first few weeks after surgery, this can be due to:

- Intraoperative drying and distortion of the flap
- Dislodgement of the flap during removal of the speculum at the end of surgery
- Rubbing of eyes by the patient
- Sleeping without topical lubricants, which may dry up the flap, especially so in patients with incomplete closure of eyelids during sleeping. The dried-up flap sticks to the eyelid and gets disturbed on waking up.

Delayed appearance of flap striae could be due to mechanical dislodgement of the flap, perhaps by a blunt trauma, and may present at any time after surgery.

### Symptoms

Blurring of vision, poor visual acuity, and monocular diplopia (double vision).

### Signs

**Figure 3:**
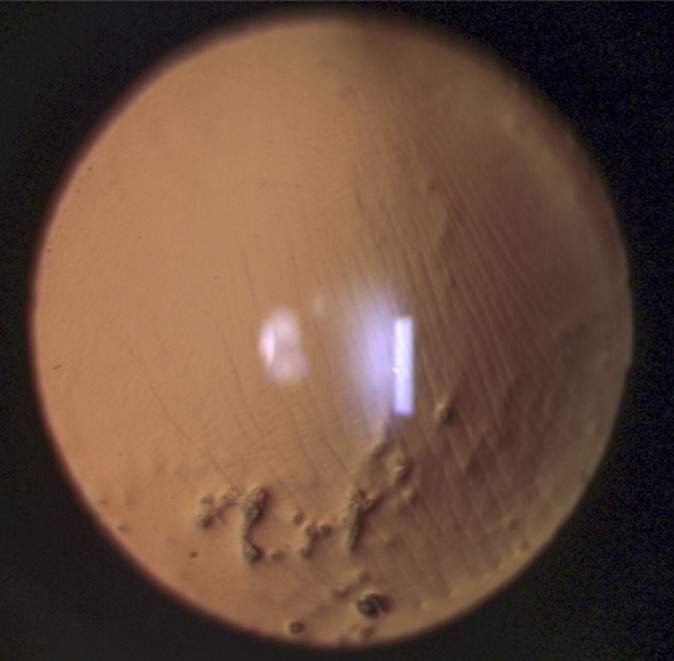
LASIK Flap macrostriae visualised with retro-illumination using a slit lamp.

Wrinkling of a LASIK flap can be seen using a slit lamp, ideally with retro-illumination (Figure 3). This can be either peripheral or finely distributed in the central portion of the flap. Disruption of the tear film may be seen after fluorescein has been put in the eye.

### Management

- **Microstriae** are fine wavy folds in the LASIK flaps, caused by fine wrinkling of the Bowman's layer and epithelium. Microstriae do not affect vision and may be observed.
- Patients with **macrostriae** have large folds on the LASIK flap. These are caused by dislocation or poor apposition of the flap. Macrostriae cause distortion of vision. In these patients, the flap needs to be lifted and repositioned.

## Raised intraocular pressure

There is a risk of the intraocular pressure (IOP) going up after laser refractive procedures, with the main cause being the use of steroid eye drops.

### Risk factors

- Frequent use of steroid eye drops, e.g., more than 4 times a day
- Patients with a history of pressure rise with steroid use
- Family history of glaucoma.

### Symptoms

- Patients may be asymptomatic, so the IOP must be monitored after the procedure, at least while the patient is on steroid drops
- Vision may be blurred if pressure-induced stromal keratitis develops (see below).

### Signs

- High IOP measurement
- A diffuse and fine stromal haze may also develop, similar in appearance to pressure-induced stromal keratitis. This is often initially diagnosed as diffuse lamellar keratitis. However, diffuse lamellar keratitis occurs earlier than pressure-induced stromal keratitis, which is usually seen more than a week after surgery.

**Figure 4:**
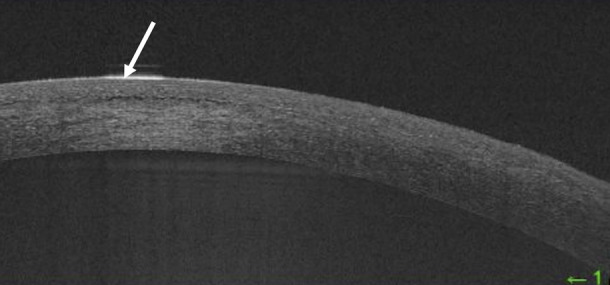
OCT of interface fluid syndrome with fluid cleft (arrow).

**NOTE:** Fluid may accumulate underneath a LASIK flap; this can cause the intraocular pressure measurement to be falsely low so that the high IOP is missed. Looking for fluid clefts with a slit section on the slit lamp is important. Fluid pockets may be seen under the flap with anterior segment OCT if available (Figure 4).

© VINEET JOSHI CC BY-NC-SA 4.0

© VINEET JOSHI CC BY-NC-SA 4.0

### Management

Corneal stromal haze that does not clear following an increased dose of topical corticosteroids should alert the physician of pressure-induced stromal keratitis. Prompt lowering of the IOP with pressure-lowering medications, shifting to low potency topical steroids, or tapering and stopping topical corticosteroids may help reduce IOP and control this condition.

## Epithelial ingrowth

Epithelial ingrowth occurs in up to 3.9% of patients receiving refractive error surgery for the first time, and in 10–20% of re-treated patients. It is more common in patients who received manual LASIK surgery.

Most symptoms appear within four weeks, although delayed presentations are not uncommon. The condition arises from the implantation of epithelial cells during surgery, or their later migration from the flap edge after surgery.

### Symptoms

Early symptoms include foreign body sensation and reoccurrence of glare due to ocular surface irregularity, while later stages may involve blurring of vision. Early epithelial ingrowth is usually hard to diagnose accurately by symptoms alone, since the symptoms may overlap with normal post-surgical discomfort.

### Clinical signs

**Figure 5:**
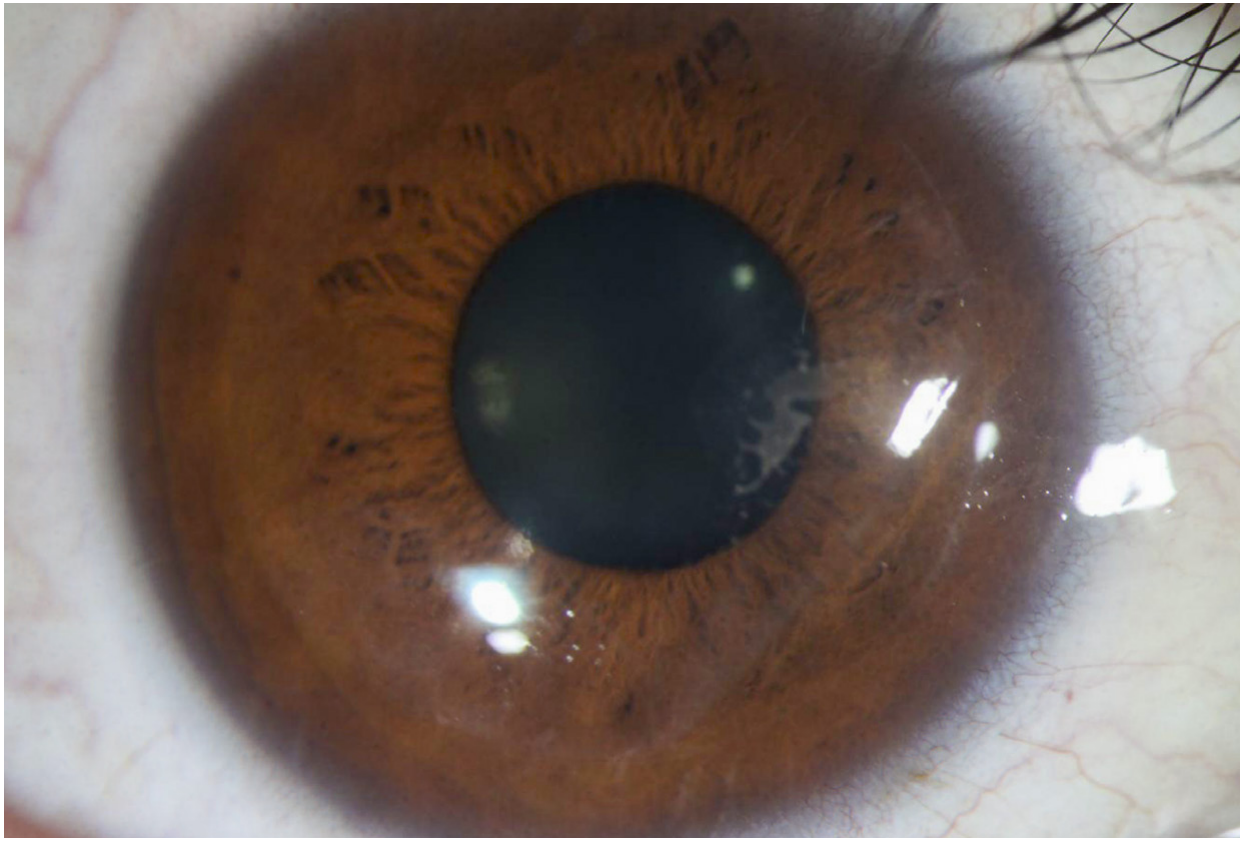
Slit lamp photography showing an area of grade 1 epithelial ingrowth with epithelial pearls.

**Figure 6:**
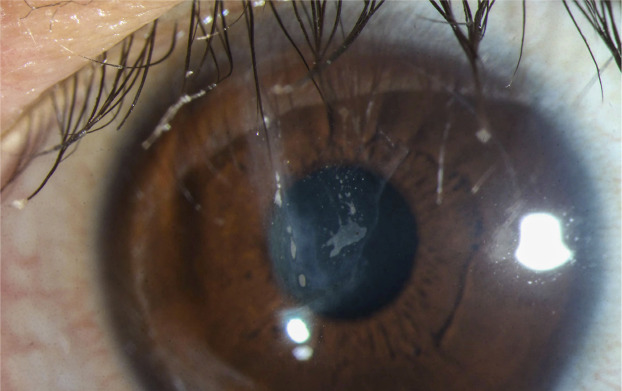
Slit lamp photography showing an area of grade 2 epithelial ingrowth with epithelial nests.

**Figure 7:**
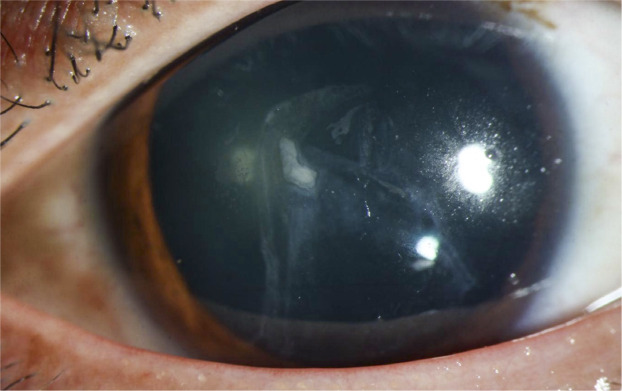
Slit lamp photography showing grades 3 to 4 epithelial ingrowth.

Slit lamp examination is more important than symptoms for early diagnosis and to grade the severity. Typical signs of epithelial ingrowth include epithelial pearls or nests (i.e., large epithelial pearls), white boundary lines along the nests at the flap-stromal interface, and flap melting (see Figures 5, 6, and 7).

© JIE YING CC BY-NC-SA 4.0

© JIE YING CC BY-NC-SA 4.0

© JIE YING CC BY-NC-SA 4.0

### Management

Patients with suspected epithelial ingrowth should be referred to an ophthalmologist who can grade the severity and begin treatment if needed. Action should be taken within 2–3 weeks, or sooner if possible.

The management of epithelial ingrowth varies according to its location, clinical features, and severity, as described in the 4-level grading system proposed by Probst and Machat.^3^ Please note that it is necessary to grade according to the most severe characteristics present to avoid delays in treatment. Detailed grading standards for each level are as follows:

- **Grade 1** epithelial ingrowth is defined as "thin growth, 1–2 cells thick, non-progressive, difficult to detect, well-delineated with a white line along the advancing edge, no flap change, and 2 mm within the flap edge.” Fortunately, since such ingrowth is not likely to be progressive, there is no treatment needed for patients with grade 1, which is the most common type seen in clinics. Figure 5 is an example of grade 1 epithelial ingrowth.
- In **grade 2**, the following characteristics (seen by slit lamp examination) would differentiate it from grade 1: thicker growth, discrete cells within an epithelial nest, no demarcation line along the nest, and an easily detectable rolled or grey flap edge with no stromal melt. Although no urgent treatment is needed, follow the patient up in 2–3 weeks; if some progression is detected, the patient should be referred to an ophthalmologist for further evaluation and treatment. Figure 6 is an example of grade 2 epithelial ingrowth.
- **Grades 3 to 4** would show much more severe clinical signs, such as pronounced/aggressive growth that is several cells thick, a rolled flap with thickened whitish-grey appearance, confluent haze at the flap edge, and flap melt beyond 2 mm from the flap edge, invading towards the visual axis. Figure 7 is an example of grade 3–4 epithelial ingrowth. Possible treatment for grades 3 and 4 include mechanical debridement, flap suturing, fibrin glue, ocular hydrogel sealant, and non-invasive neodymium-doped yttrium aluminum garnet (Nd:YAG) laser.^2^ Urgent treatment is needed.
